# Application of a Biostimulant (Pepton) Based in Enzymatic Hydrolyzed Animal Protein Combined With Low Nitrogen Priming Boosts Fruit Production Without Negatively Affecting Quality in Greenhouse-Grown Tomatoes

**DOI:** 10.3389/fpls.2022.828267

**Published:** 2022-03-02

**Authors:** Tania Mesa, Javier Polo, Andrea Casadesús, Íñigo Gómez, Sergi Munné-Bosch

**Affiliations:** ^1^Department of Evolutionary Biology, Ecology and Environmental Sciences, Faculty of Biology, University of Barcelona, Barcelona, Spain; ^2^R&D Department, APC Europe S.L., Granollers, Spain; ^3^Research Institute of Nutrition and Food Safety, Faculty of Biology, University of Barcelona, Barcelona, Spain

**Keywords:** biostimulant, cytokinins, nitrogen efficient crops, production, tomato

## Abstract

Improved nutrient use efficiency together with the use of biostimulants have been little explored thus far to improve fruit yield and quality in economically relevant crops. The aim of this study was to determine the additive or synergistic effects, if any, of the application of an enzyme hydrolyzed animal protein biostimulant (Pepton) combined with priming with low nitrogen (N) in the production and quality of greenhouse tomatoes. Biostimulant treatment (Pepton at a dose equivalent of 4 kg/ha) was applied by ferti-irrigation for 2 months during the vegetative phase both in controls (watered with nutrient solution) and nutrient efficient crop (NEC), in which plants were primed with low N by exposing them to a 30% N deficiency for 2 months, and then recovered for 1 month before fruit production. Foliar water and N contents, pigments, maximum PSII efficiency (Fv/Fm ratio), and phytohormones [including abscisic acid (ABA), salicylic acid (SA), jasmonic acid (JA), and cytokinins] were measured prior and at 4 and 8 weeks after the first application. Fruit production and quality [as indicated by total soluble sugars (TSS) and acidity (TA), and the contents of lycopene, vitamin E, and vitamin C] were measured 1 month later at harvest. Priming with low N availability (NEC plants) doubled (*p* < 0.001) fruit production (due to an increase in the number of fruits), tended to increase (*p* = 0.057) by 20% the amount of TSS and increased (*p* < 0.05) the contents of lycopene (by 90%) and vitamin E (by 40%). Pepton displayed a tendency, almost significant, to improve (*p* = 0.054) total fruit production both in control and NEC plants, thus showing an additive effect to low N priming in boosting fruit production. Pepton maintained fruit quality in terms of sugar accumulation, total acidity and the contents of carotenoids, vitamins C and E. Pepton-related improvement in fruit production seemed to be related, at least partially, to an increased accumulation of cytokinins and photosynthetic pigments in leaves, which might favor vegetative vigor and ultimately fruit yield. In conclusion, Pepton application was effective in improving the yield of greenhouse tomatoes showing additive effect with low N priming, without negatively affecting fruit quality.

## Introduction

Rising productivity to feed the escalating global population and increasing the use efficiency of the resources without harming the ecosystems are two of the greatest challenges that the agricultural sector is facing these days. Moreover, current agriculture has to face more frequent and long-lasting unfavorable environmental and soil conditions, which have a direct negative impact on crop productivity. A technological innovation proposed to solve these problems is the application of natural biostimulants that can enhance flowering, plant growth, fruit set, crop productivity, and nutrient use efficiency, while also improving abiotic stress tolerance (Rouphael and Colla, 2020). One of the main important nutrients for plant growth and development is nitrogen (N), which at the same time is one of the major limiting nutrients in most economically relevant crops (Flores et al., 2004; Padilla et al., 2018). Because of this, and to ensure high crop yield, it is very common among farmers to conduct excessive N applications that end up leaching into groundwater, threatening both the environment and human health (Padilla et al., 2018; Burri et al., 2019). Hence, it is of great importance to develop new techniques that help farmers optimize crop production, while using the minimum N input to reduce its harmful effects on the environment and human health.

Enzymatically hydrolyzed protein-based biostimulants may be an adequate approach to both raise productivity and increase the use efficiency of some nutrients such as N (Colla et al., 2014, 2017). Pepton 85/16® (Pepton) is a natural biostimulant obtained by the enzymatical hydrolysis of animal proteins that is available in a micro-granular form and highly soluble in water (APC Europe S.L., Spain). Pepton has been proved to have beneficial effects on crops under stress conditions such as intense cold or heat episodes, mild stress ambient field conditions, and water and nutrient stress. It has also been observed that it exerts a positive effect on the hormonal profile enhancing abiotic stress defenses (Polo et al., 2006; Marfà et al., 2009; Polo and Mata, 2018; Casadesús et al., 2019, 2020). However, little is still known about the possible effects of this biostimulant on plants under low nutrient availability conditions and the improvement of nutrient use efficiency. Priming with low N has been previously shown as a useful technique with a great potential to increase productivity since it can ameliorate nutrient use efficiency in wheat (Gao et al., 2019). However, to our knowledge, its potential beneficial effects on improving the yield and quality of tomatoes, and most particularly in combination with biostimulant application, has not been explored thus far.

Tomato (*Solanum lycopersicum* L.) is one of the most cultivated crops with a cultivation area of 5.03 million hectares (FAOSTAT, 2019). Its fruits are highly valued by consumers due to their organoleptic characteristics and nutritional value, as they constitute an important source of vitamins C and E and lycopene, among other nutrients and antioxidants (Asensio et al., 2019). It is of great interest to increase the nutrient use efficiency of tomato plants, without affecting their production, since large amounts of fertilizer are usually applied to obtain their maximum yield (Abenavoli et al., 2016). Therefore, this work aimed to assess to what extent an enzymatically hydrolyzed protein-based biostimulant (Pepton) can improve fruit production and quality both in control and in low N primed conditions, and establish a mechanism of action, with an emphasis on the possible role of endogenous phytohormone contents.

## Materials and Methods

### Experimental Design

Seeds of tomato plants (*Solanum lycopersicum* cv. Ailsa Craig) were obtained from the Experimental Field Facilities of the University of Barcelona (Barcelona, NE, Spain). Seeds were sown on 7th March 2019, in 1 L pots in a climate-controlled growth chamber (16 h day/8 h night, at 22°C), using a substrate based on 50% peat, 25% perlite, 25% vermiculite, CaCO_3_ at 1 g/L and essential micronutrients at 0.05 g/L. On 8th April, seedlings were transferred to 3 L pots and placed in a glass greenhouse (25.6°C mean temperature, 33.8°C highest temperature average with an absolute maximum of 41.0°C, and 60.1% mean relative humidity) with 20 cm between pots. Then, on 10th June, four treatments were established including: Control plants without Pepton, Control plants with Pepton, Nitrogen Efficient Crop (NEC) without Pepton, and NEC with Pepton. Control plants were irrigated with 50% Hoagland nutrient solution (Hoagland and Arnon, 1950) throughout the experiment. In contrast, NEC plants were primed with low N by irrigating plants for 8 weeks with a nutrient solution deficient by 30% N relative to controls and then recovered with the same nutrient solution as used for controls (50% Hoagland solution). Pepton was applied by ferti-irrigation once every 2 weeks during the first 2 months at a dose equivalent of 4 kg/ha (0.2 g of Pepton dissolved in 0.5 L of irrigation water), corresponding with the supplier recommendation for this crop. All applications were performed 1 h before sunset. All plants were subject to “topping” at week seven of starting treatments due to excessive growth in the greenhouse, so that this pruning caused the plants to stop flowering and setting new fruits.

Leaf samples were collected on 16th June (week 0), 16th July (week 4), and 13th August (week 8) at predawn (1 h before sunrise). In each sampling, one young, fully developed leaf from eight randomly selected plants per treatment was sampled. The apical leaflet was used to determine the chlorophyll fluorescence and the adjacent leaflet was used to determine the water, carbon (C), and N status, and the leaflet mass per area ratio (LMA). The other adjacent leaflet was immediately frozen in liquid N_2_ and stored at −80°C for subsequent biochemical analyses. Fruit samplings from the same plants were performed between 4th September and 7th September. All fruits (from breaker to red ripe stages) of each plant were harvested for the estimation of yield. In addition, four ripe tomatoes on the red ripe stage were chosen for fruit quality analysis: one was frozen in liquid N_2_ and stored at −80°C for subsequent biochemical analyses (total carotenoids, lycopene, and vitamins C and E) and three were used to analyze total soluble sugars (TSS) and titratable acidity (TA).

### Pepton Composition

Pepton is an enzymatically hydrolyzed protein product that contains L-α amino acids (84.8%), free amino acids (16.5%), organic-nitrogen (12.0%), iron (3,000 ppm), and potassium (4.0%). The complete chemical composition of this biostimulant can be found in Polo and Mata (2018).

### Total Production, TSS, and TA

Total production was estimated as the total amount of fruits produced per plant, the total biomass of fruits produced per plant and the fresh mass per fruit unit. The fresh mass of fruits was measured by weighing freshly harvested fruits. For estimating fruit quality, 1 ml of juice from a pool of three tomatoes was used to determine TSS using a digital refractometer HI 96801 (Hannah Instruments, Italy). Then, 10 ml of juice were diluted in 100 ml of MiliQ water and used for TA determination with 0.1 M NaOH and 1% phenolphthalein as an indicator to estimate citric acid content. The TSS/TA ratio was then calculated.

### Fruit Total Carotenoids, Lycopene, and Vitamins C and E

For the quantification of total carotenoids, 100 mg of ground frozen fruit were extracted with acetone 80% (v/v) using ultrasonication (Branson 2510 ultrasonic cleaner, Bransonic, Danbury, CT, United States). The extract was then centrifuged for 10 min at 4°C and 13,000 rpm, and the supernatant was collected. The pellet was re-extracted until it was colorless, pooling at the end all the supernatants. Total carotenoids were analyzed using UV/Visible spectrophotometry of double beam using a CE Aquarius UCE7400 (Cecil Instruments Ltd., Cambridge, United Kingdom). The absorbance of the supernatants was read at 470, 646, 663, and 750 nm, respectively. Carotenoid content was calculated following the equations developed by Lichtenthaler and Wellburn (1983).

Quantification of lycopene content was performed as described by Fish et al. (2002). Around 500 mg of ground frozen fruit were extracted with 800 μl MiliQ water, 1.5 ml acetone, 1.5 ml ethanol, and 3 ml hexane. The pellet was re-extracted with hexane until it was colorless. The absorbance of the supernatants was read at 503 and 800 nm.

The analysis of vitamin C was adapted from Takahama and Oniki (1992) and Queval and Noctor (2007). Around 100 mg of ground frozen fruit were extracted with 6% meta-phosphoric acid (w/v) and 0.2 mM DTPA using ultrasonication and centrifugation for 10 min at 4°C and 13,000 rpm. The contents of ascorbic acid (AA) and dehydroascorbic acid (DHA) were determined spectrophotometrically (xMark Microplate Spectrophotometer, Bio-Rad, Hercules, CA, United States) with quartz microplates reading the absorbance at 265 nm. Total ascorbate was calculated as the sum of AA + DHA.

For vitamin E analyses, 100 mg of ground frozen fruit were extracted with methanol and 5 ppm (w/v) of tocol as an internal standard. Extractions were performed as above. The extracts were then filtered with a hydrophobic PTFE filter of 0.22 μm (Phenomenex, Torrance, CA, United States) and injected into the HPLC system (consisting of a Waters 600 controller pump, a Waters 717 plus auto-sampler, and a Jasco FP-1520 fluorescence detector). Fluorescence detection was at an excitation wavelength of 295 nm and emission at 330 nm. The mobile phase was a mixture of *n*-hexane and 1,4-dioxane (95.5:4.5, v/v) at a flow rate of 0.7 ml/min. Tocochromanols were separated using an Inertsil 100A column (5 μm, 30 × 250 mm, GL Sciences Inc., Tokyo, Japan). A calibration curve was established with each of the tocochromanols analyzed using authentic standards from Sigma-Aldrich (Steinheim, Germany).

### Leaf Water Status, C and N Contents, and LMA

Relative water content (RWC) was calculated using the formula RWC = 100 × (FW − DW) / (TW − DW), where FW is the fresh mass measured on the sampling date, TW is the turgid mass measured after rehydrating the leaves for 24 h in the dark at 4°C, and DW is the dry mass measured after oven-drying the leaves at 80°C until constant weight. Leaflet area was measured with a flatbed scanner and processed with ImageJ (1.52p, National Institutes of Health, Bethesda, MD, United States). The LMA was calculated as DW/Leaflet area.

For C and N analyses, 3 mg of dry leaflets were ground, weighed, placed in tin capsules, sealed and analyzed using an organic elemental analyzer (Thermo EA 1108, Thermo Scientific, Milan, Italy). Standard conditions recommended by the supplier were applied (Combustion reactor at 1,000°C, reactor chromatographic column at 60°C, helium flow 120 ml/min and 10 ml oxygen circuit at 100 kPa).

### Photosynthetic Pigments and Chlorophyll Fluorescence

For total chlorophyll and carotenoid content measurements, methanolic extracts were prepared using 100 mg of frozen ground leaves and diluted 1:10 (v/v) with methanol. The absorbance of the supernatants was read at 470, 653, 666, and 750 nm using a UV/Visible spectrophotometer of double beam (CE Aquarius UCE7400, Cecil Instruments Ltd., Cambridge, United Kingdom). Chlorophyll a, chlorophyll b, and carotenoid contents were calculated following the equations developed by Lichtenthaler (1987).

The maximum efficiency of PSII photochemistry (or F*_v_*/F*_m_* ratio), an indicator of photoinhibition (Takahashi and Badger, 2011), was determined by measuring chlorophyll fluorescence from leaves by using a portable fluorimeter Mini-PAM II (Photosynthesis Yield Analyser, Walz, Germany) in dark-adapted leaves for 1 h, as described by van Kooten and Snel (1990).

### Stress-Related Phytohormones and Cytokinins

Phytohormones, including the stress-related phytohormones, abscisic acid (ABA), salicylic acid (SA), jasmonic acid (JA), and the cytokinins, *trans*-zeatin (*t*-Z), and its riboside *trans*-zeatin riboside (*t*-ZR), were analyzed by the extraction of 100 mg of frozen ground leaves with methanol containing deuterium-labeled hormones, which were used as internal standards. The extracts were subject to ultrasonication and vortexing for 30 min. After centrifugation for 10 min at 4°C and 13,000 rpm, the supernatant was collected, and the pellet was re-extracted. The supernatants were then filtered with a hydrophobic PTFE filter of 0.22 μm (Phenomenex, Torrance, CA, United States) prior to analysis. Hormone levels were analyzed by UHPLC-ESI-MS/MS as described by Müller and Munné-Bosch (2011). Quantification was made considering the recovery rates for each sample by using the deuterium-labeled internal standards.

### Statistical Analyses

Statistical analyses were performed by a three-way ANOVA for foliar analyses and a two-way ANOVA for fruit analyses. The Tuckey test was used as a *post hoc* method. In all cases, differences were considered significant at a probability level of *p* < 0.05 and a trending toward statistical significance at a probability level of *p* between 0.1 and 0.05 (Fisher, 1950; These et al., 2016). All statistical tests were performed with RStudio (Boston, MA, United States).

## Results and Discussion

### Low N Priming and Pepton Application Improve Fruit Production

**Figure 1:**
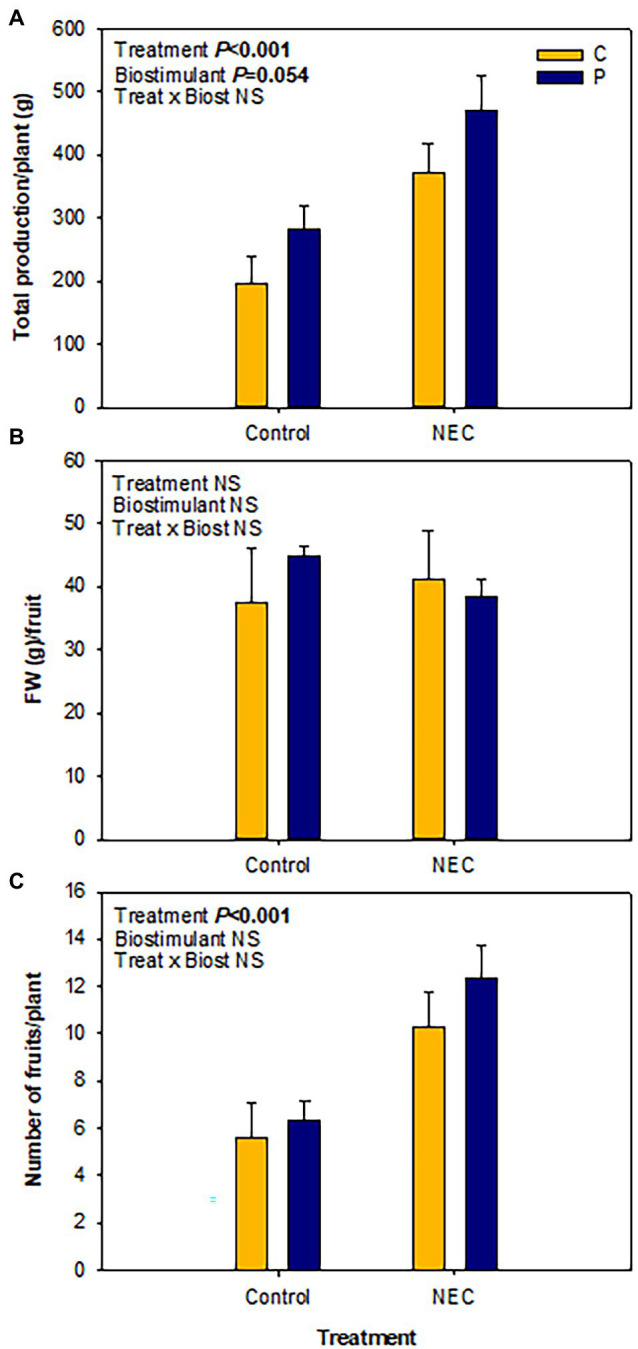
Effects of low nitrogen (N) priming and Pepton application on fruit production in greenhouse-grown tomato plants. **(A)** Total fruit production per plant, **(B)** average fresh weight of ripen fruits, and **(C)** number of ripen fruits per plant, in Pepton-treated plants (P) or untreated controls (C) either fed with nitrogen in a 50% Hoagland solution (Control) or exposed to low nitrogen priming (NEC). NEC stands for Nitrogen Efficient Crop (these plants were exposed to a 30% N deficiency relative to controls for 2 months and then recovered for 1 month with the same nutrient solution used for controls before estimation of fruit production, see section Materials and Methods for details). Data are mean of *n* = 8 ± SE. *p* values of two-way ANOVA are shown in the inlets and values of *p* > 0.10 were considered as not significant (NS).

Biostimulants have been considered innovative tools that are able to improve plant growth and productivity and help alleviate the effects of abiotic stresses. Furthermore, some biostimulants, such as Pepton, that are enzymatically hydrolyzed animal protein-based biostimulants can promote circular economy helping recycle animal waste products and reduce the use of limited resources of nature (Casadesús et al., 2020). Previous studies have shown that Pepton can improve crop performance against different abiotic stresses including water and temperature stress conditions. In the present study, fruit production was stimulated by the NEC treatment as compared to controls, whereas fruit size remained unaffected (Figure 1). NEC treatment increased the production of greenhouse-grown tomato fruits, estimated as total fresh matter of fruits per plant, by 1.7-fold, which was associated with an increase in the number of fruits per plant by 1.8-fold. However, no significant differences were observed for fruit size. A trend toward statistical significant effect (*p* = 0.054) was observed for fruit production in Pepton-treated plants, with an increase in fruit production by 32% relative to controls both in N-fed (Controls) and low N-primed plants (NEC; Figure 1). This increase in yield in both groups of plants supplemented with Pepton has an important economic relevance for producers with very positive return on investment. In a previous study, Polo and Mata (2018) found similar results, with a yield increased by 27% when Pepton was applied at similar doses (4 Kg/Ha) in gold cherry tomatoes growing under low stress ambient field conditions. N is a crucial nutrient for plant growth and development, so one might expect that plants with higher N availability (control, N-fed plants) would show increased fruit production, as previously observed in several economically important crops, including tomatoes (Warner et al., 2004). It is also true, however, that low N priming can be used in crops to improve nutrient efficiency, showing a great potential to increase yields (Gao et al., 2019). In the present study, we observed that plants have acclimated to a deprived nutrient availability during the vegetative growth for 2 months allowing them to become more efficient in their N use and, therefore, increase their total fruit production considerably. It is noteworthy, however, that aside from low N priming, the pruning of plants performed after 7 weeks of treatment may play a role in the observed effects. Topping might have caused a severe loss of aboveground biomass favoring fruit set and ripening, but at the same time reduced a significant amount of biomass previously consuming root N. Therefore, as pruning was performed just before the end of N deprivation, it may have had an impact on the recovery phase in the NEC treatment helping boost production relative to controls. In other words, NEC treatment may would not have had such a positive effect compared to controls if all plants had not been pruned.

### The Positive Effects of Combined Low N Priming and Pepton Application on Fruit Production Did Not Negatively Affect Fruit Quality

**Figure 2:**
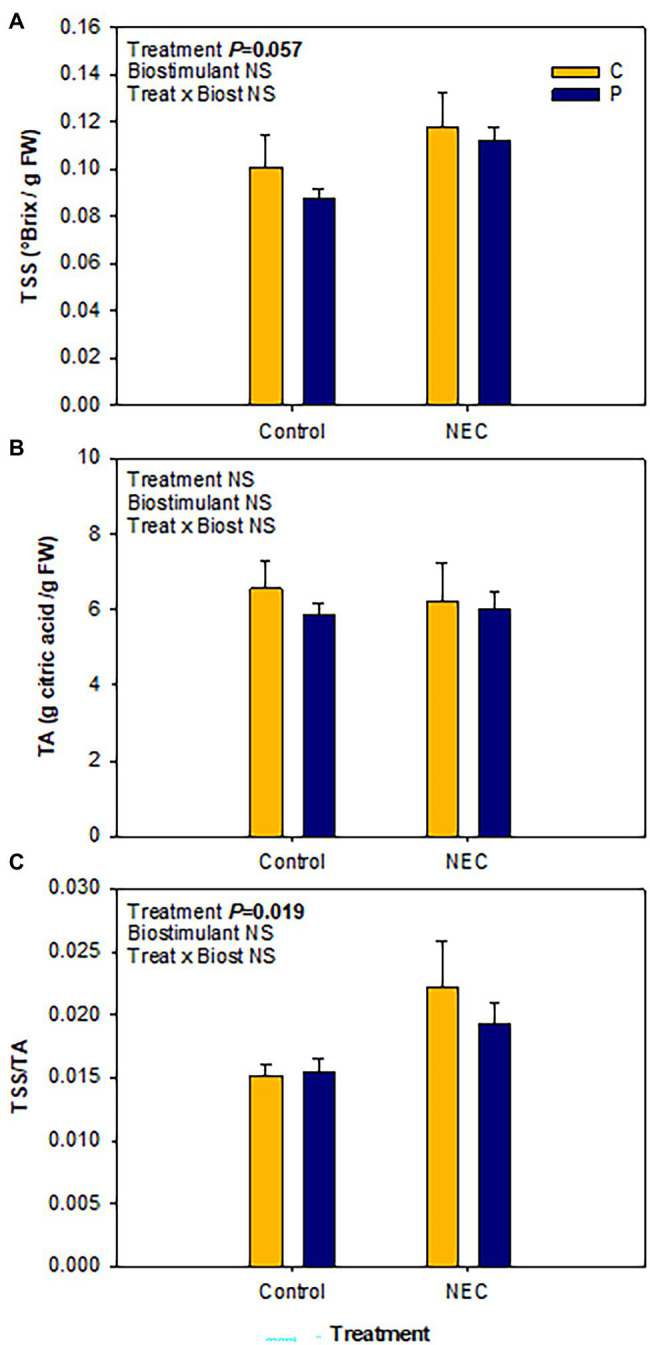
Effects of low nitrogen priming and Pepton application on fruit quality, in terms of soluble sugar accumulation and acidity, in greenhouse-grown tomato plants. **(A)** Total soluble sugars (TSS), **(B)** total acidity (TA), and **(C)** TSS/TA ratio in mature fruits of Pepton-treated plants (P) or untreated controls (C) either fed with nitrogen in a 50% Hoagland solution (Control) or exposed to low nitrogen priming (NEC). NEC stands for Nitrogen Efficient Crop (see Figure 1 legend and section Materials and Methods for details). Data are mean of *n* = 8 ± SE. *p* values of two-way ANOVA are shown in the inlets and values of *p* > 0.10 were considered as NS.

**Figure 3:**
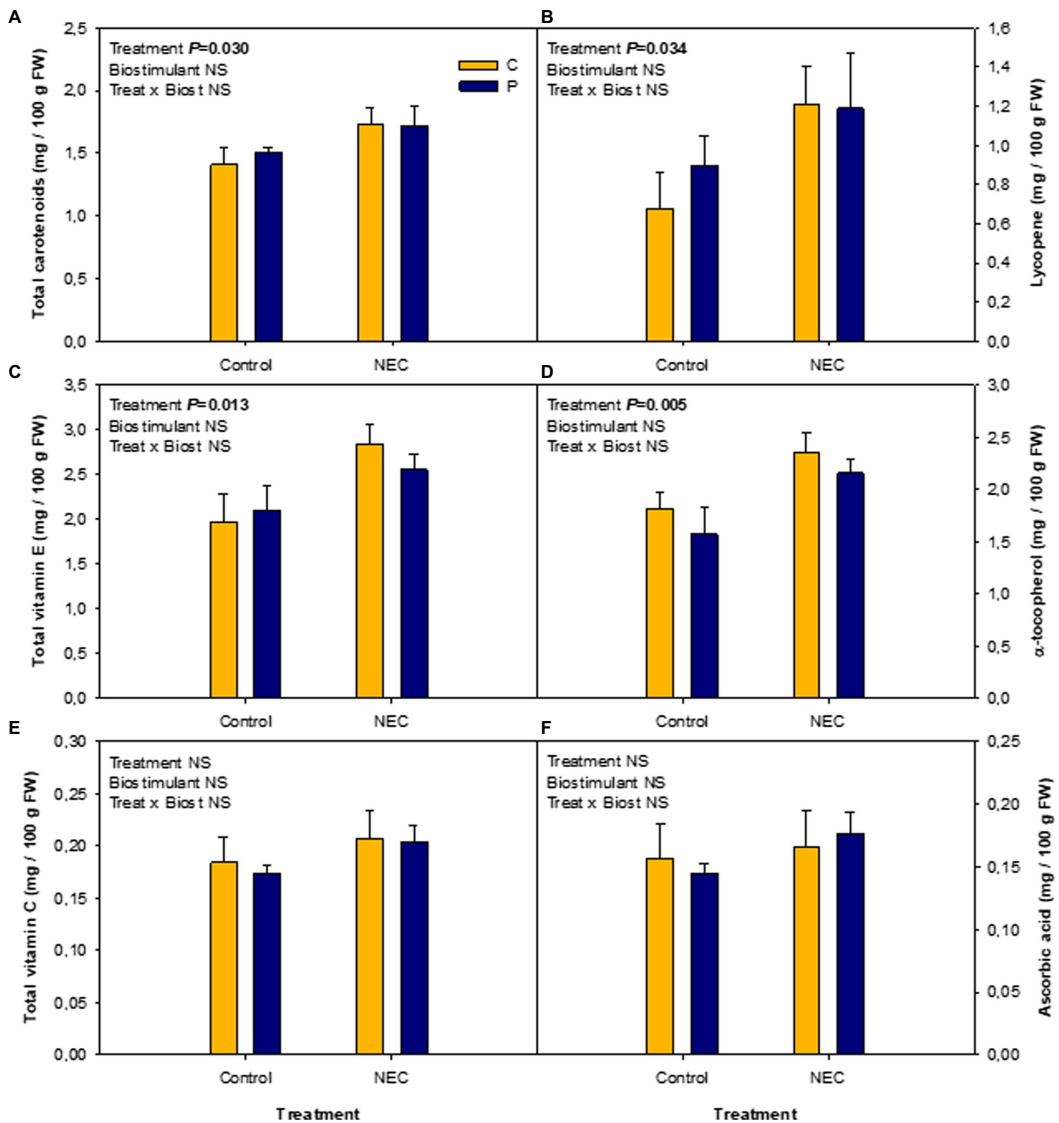
Effects of low nitrogen priming and Pepton application on fruit quality, in terms of carotenoid, vitamin E, and vitamin C accumulation, in greenhouse-grown tomato plants. **(A)** Total carotenoids, **(B)** lycopene, **(C)** total vitamin E, **(D)** α-tocopherol, **(E)** total vitamin C, and **(F)** ascorbic acid contents in mature fruits of Pepton-treated plants (P) or untreated controls (C) either fed with nitrogen in a 50% Hoagland solution (Control) or exposed to low nitrogen priming (NEC). NEC stands for Nitrogen Efficient Crop (see Figure 1 legend and section Materials and Methods for details). Data are mean of *n* = 8 ± SE. *p* values of two-way ANOVA are shown in the inlets and values of *p* > 0.10 were considered as NS.

Total soluble sugars and TA are important components of flavor exerting their effect not only through their content but also through their ratio (Xu et al., 2018; Navarro and Munné-Bosch, 2022). However, the effects of some nutrients’ availability, such as a decrease in the N dose, on tomato quality are still controversial as it has been seen to both increase (Aziz, 1968; Bénard et al., 2009; Truffault et al., 2019) and decrease (Hernández et al., 2020; Navarro and Munné-Bosch, 2022) the TSS/TA ratio. In the present study, fruit quality was improved by the NEC treatment, particularly, a significant increase in the TSS/TA ratio was observed together with a trend toward statistical significant increase of TSS (Figure 2). Moreover, the quality of the tomato fruits was not affected by the application of Pepton, thus showing that this biostimulant can increase fruit yield without a negative impact on the TSS/TA ratio. Other quality parameters related to the antioxidant composition of the fruits, were also unaffected by the biostimulant, again indicating that it would be a great option to increase the production without negative implications on the fruit quality (Figure 3). Furthermore, NEC treatment did improve the total carotenoids and vitamin E contents by increasing their major components, lycopene, and α-tocopherol, respectively. Total carotenoids and lycopene contents increased by NEC treatment by 18 and 52%, respectively, relative to controls. Vitamin E in fruits also increased in response to the NEC treatment by 33% relative to the controls, mainly associated with an increase of α-tocopherol (Figure 3). In contrast, total vitamin C and ascorbic acid contents remained unaffected by treatments.

### Effects of Low N Priming and Pepton Application on Fruit Production Differ on a Mechanistic Level

**Figure 4:**
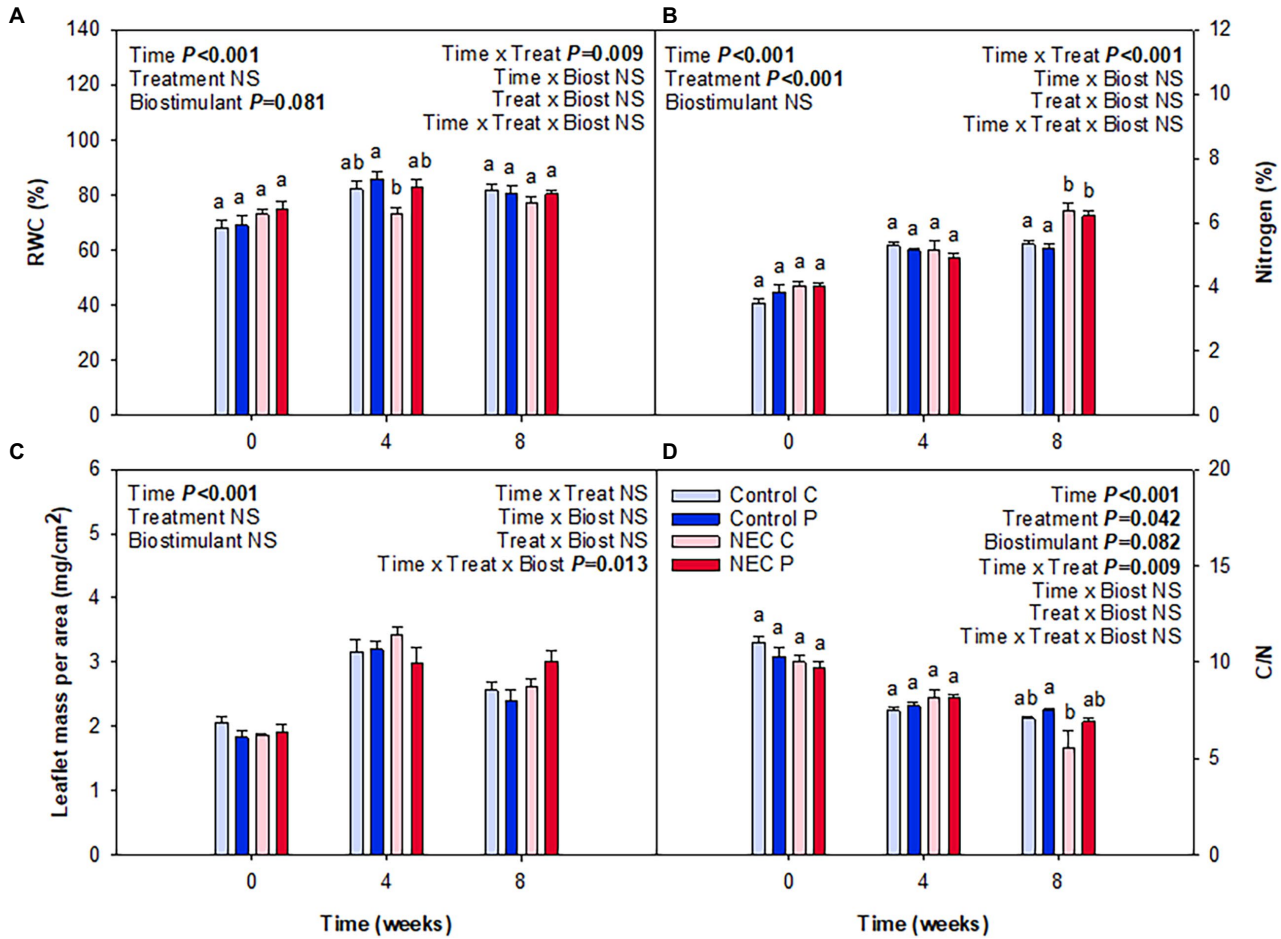
Effects of low nitrogen priming and Pepton application on leaf water and nitrogen contents in greenhouse-grown tomato plants. **(A)** Relative leaf water content (RWC), **(B)** nitrogen content (expressed as a percentage of dry mass), **(C)** leaflet mass per area ratio (LMA), and **(D)** carbon to nitrogen (C/N) ratio. NEC stands for Nitrogen Efficient Crop (see Figure 1 legend and section Materials and Methods for details). Measurements on leaves were performed before treatments (week 0), and in the middle (week 4) and end (week 8) of treatments. Data are mean of *n* = 8 ± SE. *p* values of three-way ANOVA are shown in the inlets and values of *p* > 0.10 were considered as NS. Different letters indicate differences in Tukey’s HSD multiple comparison test.

**Figure 5:**
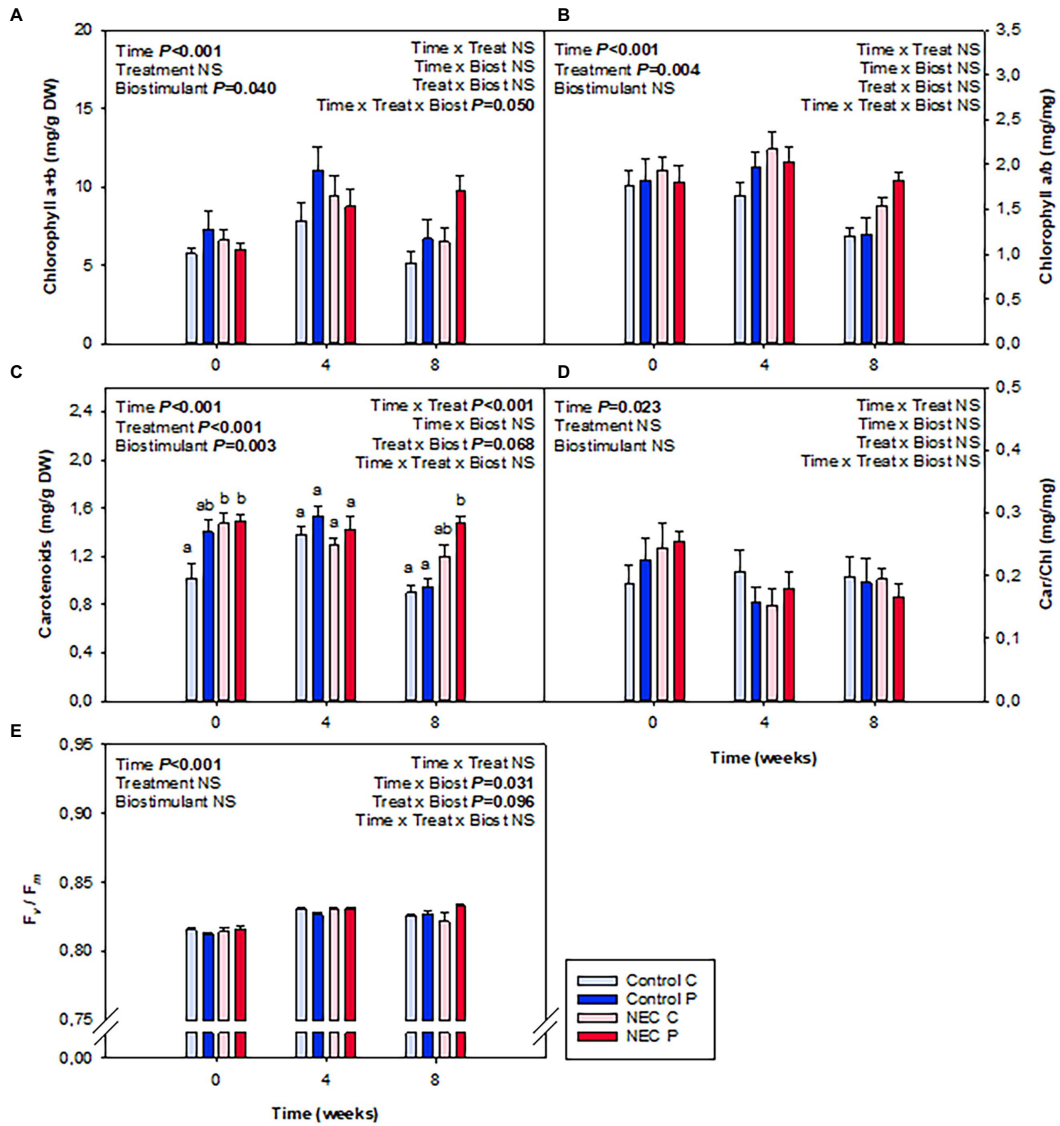
Effects of low nitrogen priming and Pepton application on photosynthetic pigment contents and photoinhibition in leaves of greenhouse-grown tomato plants. **(A)** Chlorophyll a + b, **(B)** chlorophyll a/b ratio (Chl a/b), **(C)** carotenoids, **(D)** carotenoids/chlorophylls ratio (Car/Chl), and **(E)** maximum efficiency of photosystem II photochemistry (*F*_v_*/F*_m_), an indicator of photoinhibition. NEC stands for Nitrogen Efficient Crop (see Figure 1 legend and section Materials and Methods for details). Measurements on leaves were performed before treatments (week 0), and in the middle (week 4) and end (week 8) of treatments. Data are mean of *n* = 8 ± SE. *p* values of three-way ANOVA are shown in the inlets and values of *p* > 0.10 were considered as NS. Different letters indicate differences in Tukey’s HSD multiple comparison test.

**Figure 6:**
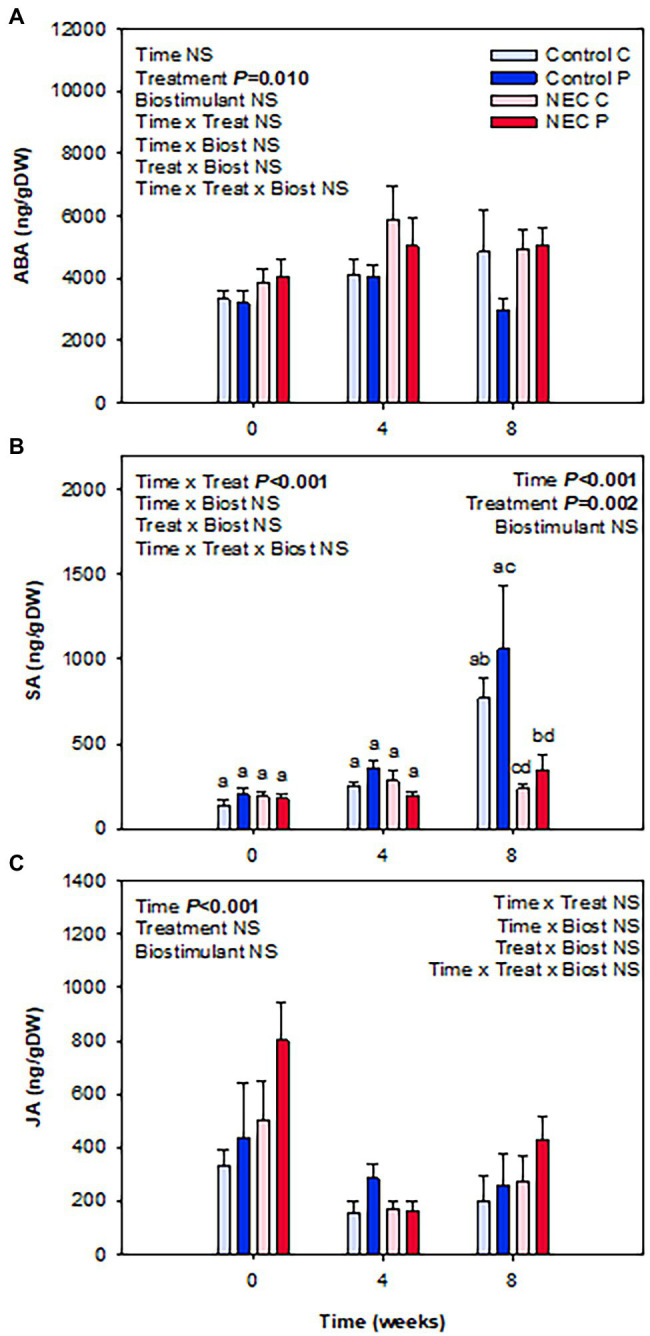
Effects of low nitrogen priming and Pepton application on the endogenous contents of stress-related phytohormones in leaves of greenhouse-grown tomato plants. **(A)** Abscisic acid (ABA), **(B)** salicylic acid (SA), and **(C)** jasmonic acid (JA). NEC stands for Nitrogen Efficient Crop (see Figure 1 legend and section Materials and Methods for details). Measurements on leaves were performed before treatments (week 0), and in the middle (week 4) and end (week 8) of treatments. Data are mean of *n* = 8 ± SE. *p* values of three-way ANOVA are shown in the inlets and values of *p* > 0.10 were considered as NS. Different letters indicate differences in Tukey’s HSD multiple comparison test.

**Figure 7:**
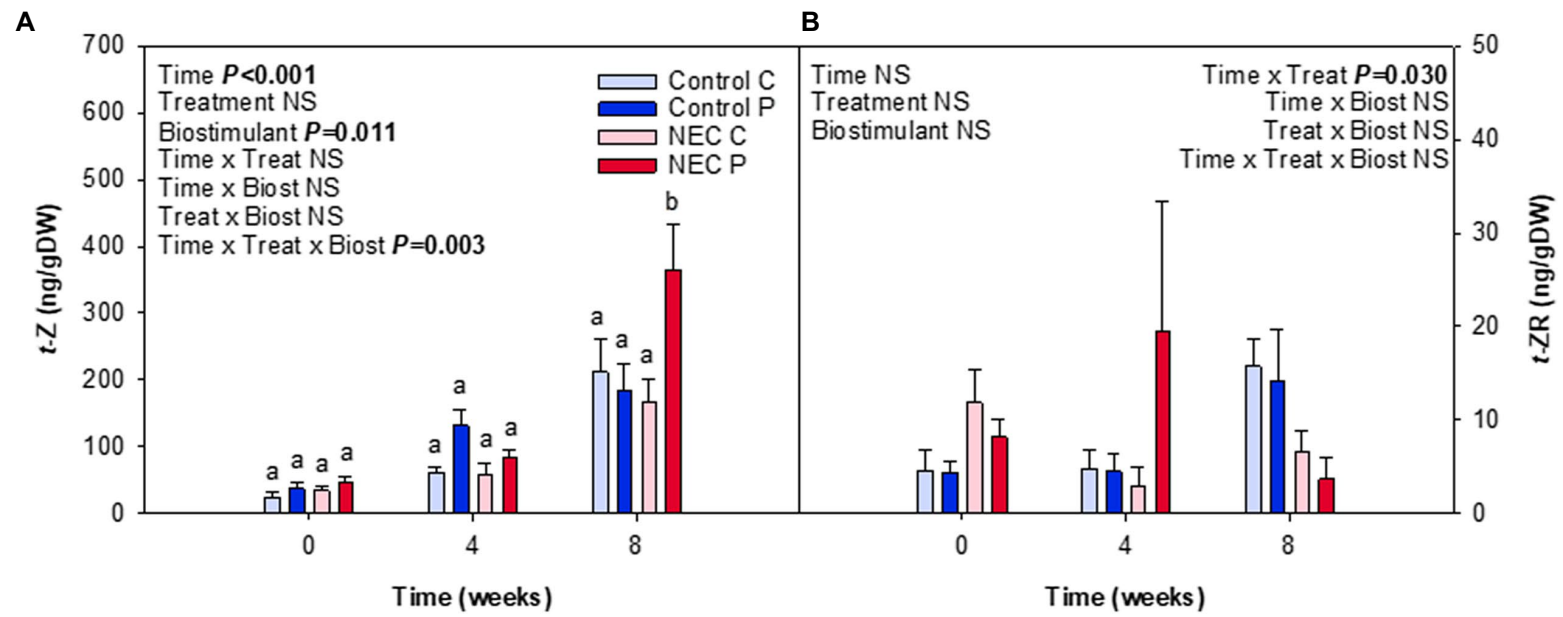
Effects of low nitrogen priming and Pepton application on the endogenous contents of cytokinins in leaves of greenhouse-grown tomato plants. **(A)** *trans*-zeatin (*t*-Z) and **(B)** *trans*-zeatin (*t*-ZR). NEC stands for Nitrogen Efficient Crop (see Figure 1 legend and section Materials and Methods for details). Measurements on leaves were performed before treatments (week 0), and in the middle (week 4) and end (week 8) of treatments. Data are mean of *n* = 8 ± SE. *p* values of three-way ANOVA are shown in the inlets and values of *p* > 0.10 were considered as NS. Different letters indicate differences in Tukey’s HSD multiple comparison test.

When analyzing water and N contents in leaves, it was observable that a significant increase of N content occurred due to the NEC treatment, especially at week 8 of treatment. This increase was reflected in the percentage of total N and the C/N ratio (Figure 4). Pruning during week 7 may have caused this effect. Despite plants suffering 30% N deficiency, the removal of a significant part of the aerial biomass may have largely alleviated stress and have led to an improvement of N use in all treatments, but most particularly in those already suffering a N deficiency. Differences between NEC and control treatments were small but significant, with an increase in foliar N by 15% in the former compared to the latter. In contrast, Pepton treatment did not additionally increase foliar N contents, but showed a trend to improve the RWC (*p* = 0.081), an effect that appeared to be more apparent before than after pruning (week 4 relative to week 8; Figure 4). The contents of photosynthetic pigments were also positively affected by either the NEC treatment, the biostimulant or both, depending on the parameter (Figure 5). The most striking effects were observed for the biostimulant combined with the NEC treatment. Pepton application significantly helped to maintain the contents of chlorophyll a + b and total carotenoids higher than in controls, an effect that was particularly observed at week 8 in NEC plants treated with Pepton (Figure 5). On the other side, NEC treatment alone helped maintain carotenoids content and the chlorophyll a/b ratio high at week 8, but not as high as when combined with Pepton (Figure 5). This Pepton-related differential behavior in NEC plants may be attributed to an increased endogenous concentration of cytokinins, and most particularly of the active form *t*-Z, but not of any of the stress-related phytohormones, ABA, SA, or JA (Figures 6, 7). NEC treatment had an impact on the endogenous content of ABA, which was most evident at week 4, but most particularly in the endogenous content of SA at week 8 (Figure 6). As SA is a well-known growth inhibitor (Abreu and Munné-Bosch, 2009), reduced contents of SA in leaves of NEC may contribute to enhanced yields in low N-primed plants, an effect that occurred both with and without Pepton application. In contrast, Pepton doubled the endogenous contents of *t*-Z in combination with the NEC treatment (Figure 7), thus showing a possible link with the maintenance of chlorophyll contents (Figure 5). It is well-known that high cytokinins contents may have an anti-senescing effect and help maintain the photosynthetic apparatus active for longer (Müller and Munné-Bosch, 2021), which in this case might contribute to increase fruit yield in greenhouse-grown tomatoes subject to a combination of Pepton application and low N priming. It is interesting to note that this cytokinin-mediated effect was not observed for NEC only, thus indicating differential mechanisms of action upon the application of Pepton. It was only the combination of low N priming together with Pepton application that caused additive effects on yield, enhanced cytokinin contents and a maintenance of chlorophyll contents. It is tempting to speculate that the composition of peptides in Pepton may cause such an effect, since previous studies have shown a crosstalk between peptide and cytokinin signaling in plants (Leibfried et al., 2005; Fukuda and Higashiyama, 2011; Sakakibara, 2021), an aspect that warrants further investigations.

Another important point that needs consideration is the application and interpretation of statistical evaluation of results in the study of the application of biostimulants to improve crop yields and produce quality, either in the field or in controlled trials, as that performed in the present study. Statistical analyses generally rely on *p* values to demonstrate relationships and the traditional level of significance (*p* < 0.05) can indeed be negatively impacted by small sample size, bias, and random error, so that in psychology and biochemical research has sometimes evolved to include interpretation of statistical trends, correction factors for multiple analyses, and acceptance of statistical significance for *p* > 0.05 for complex relationships such as effect modification (These et al., 2016). We suggest that in very particular cases, where biostimulants may have relatively effects by themselves but they can indeed modify the effect of another treatment, as it occurred in our study with low N priming, it is suggested to consider *p* values close to 0.05 as showing a trend to significant effect and must not be disregarded. As shown in the present study, these effects may be out of the classical *p* value but at the same time represent important increases in yield that if confirmed under field conditions will lead to important economical revenues. It is therefore of value to include results with a trend to statistically significant results, so that these positive effects can be scalable and therefore adequately tested in the field in future studies. Furthermore, we strongly encourage research that relies on various yield and quality parameters, instead of using only one parameter, since any small unavoidable bias due to the intrinsic methodology used on the latter case can lead to equivocal interpretation of results.

## Conclusion

It is concluded that priming with low N availability (NEC plants) doubled fruit production (due to an increase in the number of fruits), increased by 20% the amount of total soluble sugars and increased the contents of lycopene (by 90%) and vitamin E (by 40%). Furthermore, it is also stated that pruning the plants may have helped NEC treatment boost the production since there was a reduction of the root N-consuming biomass. Pepton treatment showed a trend to improve total fruit production both in control and NEC plants, thus indicating an additive effect to low N priming in boosting fruit production. Pepton maintained fruit quality in terms of sugar accumulation, total acidity, and the contents of carotenoids, vitamins C and E. Pepton-related improvement in fruit production seemed to be related, at least in part, to an increased accumulation of cytokinin and photosynthetic pigments in leaves, which might favor vegetative vigor and ultimately fruit yield. It should be noted that the observed effects may at least be partially attributed to the pruning that was carried out 7 weeks after treatment, thus it imperative to carry out future research to unravel the mechanisms underlying the interactive effects of low N priming with biostimulant application and pruning.

## Data Availability Statement

The original contributions presented in the study are included in the article/supplementary material, further inquiries can be directed to the corresponding author.

## Author Contributions

JP and SM-B conceived and designed the experiments with the help of AC. AC, IG, and TM performed the experiments. AC and TM prepared the figures and performed the statistical analyses. SM-B wrote the first draft of the manuscript with the help of TM and JP. All authors contributed to the article and approved the submitted version.

## Funding

This study was partially funded by the European Union Regional Development Fund within the framework of the ERDF Operational Program of Catalonia 2014–2020 with the reference RD17-1-0096 (Generalitat de Catalunya, Spain) and by APC Europe S.L. The funders were not involved in the study design, collection, analysis, interpretation of data, the writing of this article, or the decision to submit it for publication.

## Conflict of Interest

JP is employed by APC Europe S.L.

The remaining authors declare that the research was conducted in the absence of any commercial or financial relationships that could be construed as a potential conflict of interest.

## Publisher’s Note

All claims expressed in this article are solely those of the authors and do not necessarily represent those of their affiliated organizations, or those of the publisher, the editors and the reviewers. Any product that may be evaluated in this article, or claim that may be made by its manufacturer, is not guaranteed or endorsed by the publisher.
